# Advancing Auricular Reconstruction: The Evolution and Outcomes of Auricular Reconstruction Using a Porous Polyethylene (PPE) Framework

**DOI:** 10.3390/jcm14124116

**Published:** 2025-06-10

**Authors:** Sara M. Hussein, Basel A. Sharaf, Samir Mardini, Waleed Gibreel

**Affiliations:** 1 Division of Plastic and Reconstructive Surgery, Department of Surgery, Mayo Clinic, Rochester, MN 55905, USA; hussein.sara@mayo.edu (S.M.H.);; 2 Department of Pediatric and Adolescent Medicine, Mayo Clinic, Rochester, MN 55905, USA

**Keywords:** craniofacial, ear deformities, operative techniques, biocompatible materials, three-dimensional printing, esthetic surgery

## Abstract

**Background/Objectives** Auricular reconstruction poses significant surgical challenges in congenital and post-traumatic cases. Porous polyethylene (PPE) implants have emerged as a biocompatible alternative to the traditional autologous rib cartilage frames, offering less morbidity and a potentially stable framework. Here, we summarize the current evidence of the use of PPE auricular implants. **Methods**: A literature search was performed in accordance with PRISMA guidelines across several databases. Studies reporting outcomes of PPE implants in auricular reconstruction were included. Data were extracted on patient characteristics, operative details, and complication rates, along with any required interventions to address complications. Complications were classified as minor or major based on their management strategy. **Results**: Of 544 screened studies, 14 studies representing 1036 patients were included. PPE implant use was generally linked with favorable esthetic outcomes and high patient satisfaction (80%). Study-to-study variation in complication rates was notable, with some complication rates as high as 44% in the early 1990s. By the early 2000s, advancements in surgical methods—particularly the use of temporoparietal fascia (TPF) flaps and other flaps for optimal soft tissue coverage—had markedly reduced complication rates, with recent studies reporting rates as low as 7%. Implant exposure (6.7%) and implant fractures (ranging from 1.6% to 3.2%) were the most frequently reported problems. **Conclusions**: PPE auricular implants, despite decades of availability, have faced limited global adoption due to concerns over complications and longevity. Advances in surgical techniques have significantly reduced complication rates (<7%), making PPE implants a viable early intervention with favorable esthetics and negligible donor-site morbidity.

## 1. Introduction

Auricular reconstruction is a complex and challenging area in the field of plastic and craniofacial surgery, especially in the management of congenital and traumatic auricular deformities [1,2,3,4]. The restoration of a normal-appearing and esthetically pleasing auricle is the goal of every auricular reconstructive procedure [2]. Although microtia is a relatively rare congenital condition with an incidence rate of about 1 in 3000 to 20,000 live births globally [4], it is not uncommon in reconstructive clinical practice [1,5]. The surgical treatment of microtia is quite complex for several reasons. Such complexity lies mostly in the creation of a durable framework and provision of reliable soft tissue coverage [1,5]. Traditional methods have relied heavily on autologous grafts, particularly rib cartilage, which provides reliable structural support. However, the use of autologous cartilage grafts has notable drawbacks [1,6,7], such as the need for several surgical procedures and donor site morbidities [4,8]. The procedure is often performed in older children (minimum age of 8–10 years), and the durability and long-term outcomes can be unpredictable [4,8]. The quest for less invasive, more efficient and durable alternatives has been the driving force behind advancements in alloplastic auricular reconstruction [9].

Since the mid-20th century, more than 40 distinct materials have been reported for use in creating auricular frameworks, with the objective of enabling repair at an earlier stage than conventional cartilage harvesting. Therefore, early auricular reconstruction with alloplastic implants is attempted to reduce the social stigma and psychological impact that uncorrected auricular deformities may cause during childhood and school years [10,11,12]. Alloplastic materials like ivory, silicone, metal, rubber, acrylate, Teflon, and porous polyethylene (PPE) have been explored for their potential in restoring the auricular framework [1]. Specifically, the introduction of PPE implants, commonly known as MEDPOR™, marked a pivotal shift in the biocompatible reconstructive options available to surgeons. In 1982, Dr. Alexander Berghaus reported the earliest application of PPE implants for auricular reconstruction [1,4]. This was followed by Wellisz in 1993 [2], demonstrating the potential of the PPE implants to address more complex auricular deformity cases. The PPE’s unique porous structure supports the ingrowth of host tissue and collagen deposition, creating a stable and integrated implant [5,9,13]. This procedure was further refined, developed, and popularized by Dr. John Reinisch [12]. Still, these materials often provide challenges including implant extrusions, exposures, infections, and fractures.

This study sought to provide a comprehensive overview of PPE in auricular reconstruction from its early use to current evidence supporting its benefits, and the reported outcomes.

## 2. Materials and Methods

### 2.1. Search Strategy and Study Selection

A comprehensive literature search was conducted across several databases, following the Preferred Reporting Items of Systematic Review and Meta-Analysis (PRISMA) guidelines. The study selection process is summarized in the PRISMA flowchart (Supplementary Figure S1). This search identified studies on auricular reconstruction using PPE implants. The search included all studies published from inception through December 2024. The search strategy utilized the following keywords: “Ear” OR “Auricle” AND “Porous Polyethylene” OR “PPE.” The inclusion criteria were the use of PPE auricular implants in clinical patients, and detailed patient outcomes, complications, and proper follow-up. Case reports and case series were also included in this review if the proper follow up was reported. Studies were excluded if the full article was unavailable, if they were cadaveric or animal studies, if they lacked specific patient data or outcome measures, or had no accessible English translation. Three hundred articles were excluded before the full text review step.

### 2.2. Data Collection and Analysis

Data were extracted on patient demographics, surgical technique details, including the use of tissue expanders, different PPE frameworks, flap selection and soft tissue coverage, and the stages of the operative procedures. All complications were systematically categorized and analyzed. We aimed to standardize the reporting of surgical outcomes, complications, and long-term follow-up. Therefore, infection cases were grouped into two distinct categories: those successfully treated without implant removal and those necessitating implant removal. Similarly, cases of implant exposure were classified into two subcategories: exposure managed with local flap coverage and exposure that required implant removal. Importantly, in cases where multiple studies reported outcomes from the same patient cohort under the same senior author or research group, the study with the largest and most comprehensive patient pool was selected to ensure the inclusion of the most representative outcome data for our analysis. This review provides a descriptive analysis of outcomes related to PPE-based auricular reconstruction over decades of use. Due to the limited number of clinical integrations of PPE auricular implants and overlapping patient cohorts, a meta-analysis was not feasible.

## 3. Results

### 3.1. Included Studies and Origin Countries of Publication

**Figure 1:**
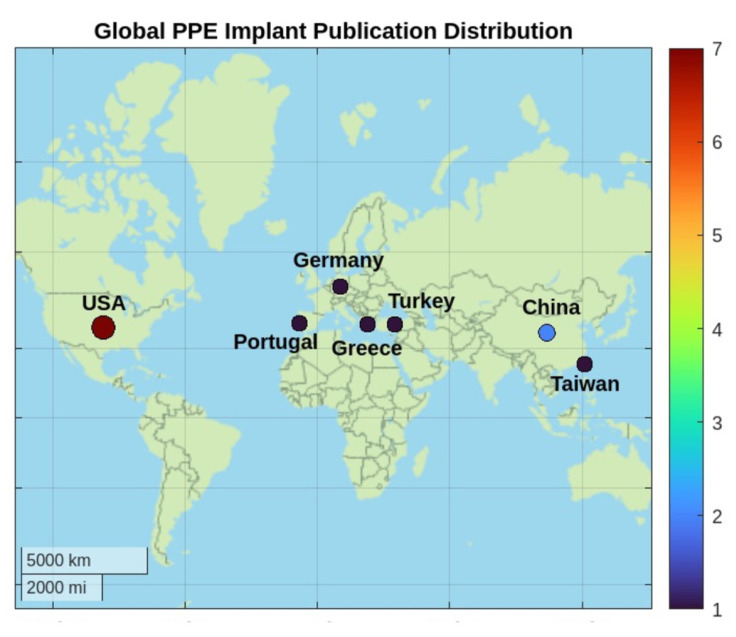
Countries with publications reporting outcomes of porous polyethylene implants in auricular reconstruction. Marker size and color scale both represent publication volume, with red indicating high volume and black to blue indicating lower volume.

A total of 544 studies were identified in the initial search. After removing duplicates (*n* = 152) identified by Covidence, 392 unique studies remained. Titles and abstracts of these studies were screened for relevance. Of the remaining 85 studies sought for retrieval, 74 were excluded after full-text review due to various reasons, including inadequate data reporting, irrelevant intervention, or lack of significant reported outcomes. Additionally, studies with suspected overlapping patient pools by the same lead or senior author were also excluded. Moreover, we added some studies from the citations of the included articles. A total of 14 studies on PPE auricular reconstruction met the inclusion criteria, representing around 1718 ears for 1036 patients. The included studies were conducted in various countries, including USA, China, Germany, Turkey, Taiwan, Portugal, and Greece (Figure 1).

In North America and Europe, one-stage reconstruction is the most frequently used technique. If necessary, the second procedure is reserved for any additional refinements. This one-stage approach is praised for minimizing the overall surgical burden on children, allowing for earlier reconstruction and avoiding chest morbidities that are associated with the conventional cartilage techniques [8,12,14,15,16,17,18,19]. The United States has led the one-stage PPE auricular reconstruction, highlighting the benefits of early intervention and superior esthetic outcomes in comparison to the conventional options. On the other hand, China and Taiwan reported the two-stage approach involving tissue expansion prior to PPE auricular reconstruction [3,20].

Despite this variation in surgical approach, the majority of the studies emphasized the significance of interdisciplinary care in auricular reconstruction patients to restore both the function and esthetics of the auricle. In patients with anotia or microtia, the deformity can extend beyond the absent or hypoplastic auricle to include varying degrees of external auditory canal atresia, middle auricle malformation, and varying degrees of hearing loss. Therefore, the multidisciplinary approach is crucial to ensure comprehensive assessment and the creation of an individualized treatment approach.

### 3.2. Patient Demographics

A total of 1718 auricles in 1036 patients were reported across the 14 studies. Most studies were retrospective in nature, with seven retrospective cohort studies, five case reports, and one case series. Only two prospective studies were reported. Patient demographics are summarized in Table 1. The average age for congenital deformity reconstruction was 8.2 years old (ranging from 3 to 14 years old), and the average age for adult reconstruction was 31.7 years old (ranging from 20 to 59 years old). For instance, Reinisch et al. reported that PPE auricular reconstruction can be performed in children as young as 2 years and 8 months [12]. Gender representation was mostly female (64%). The follow-up duration varied across studies from 6 months to 19 years, with a median of 1.9 years.

### 3.3. Surgical Indications and Staging

#### One-Stage Approach

**Figure 2:**
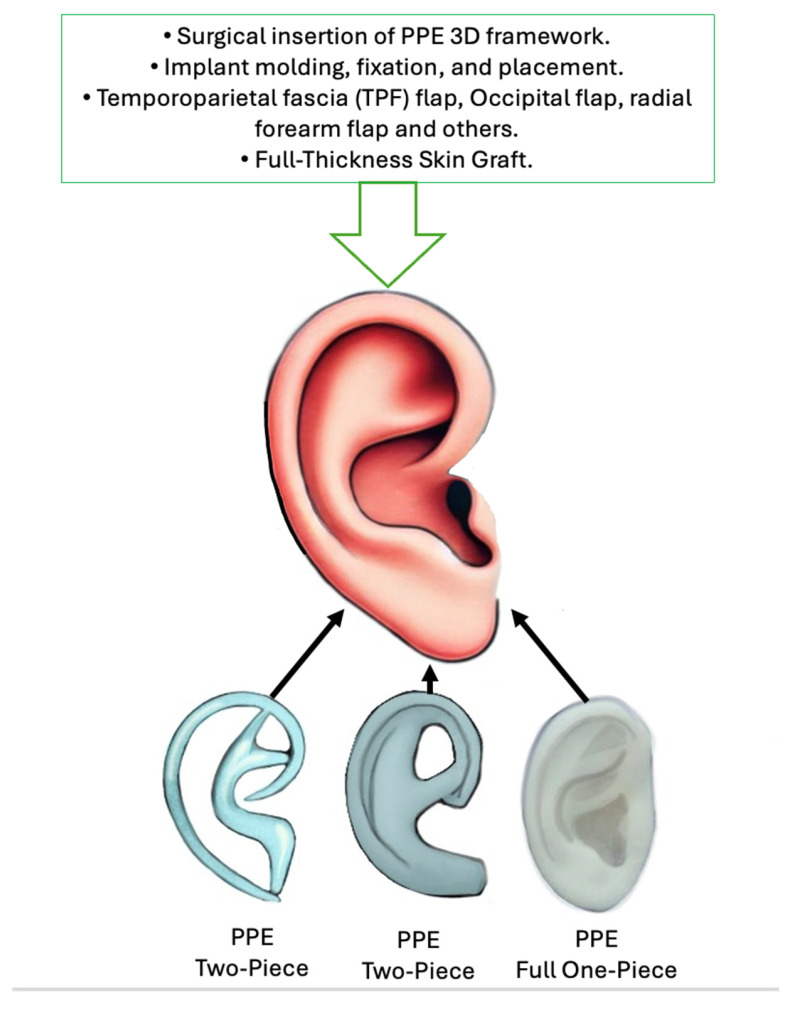
Summary of the one-stage PPE auricular reconstruction procedure and the evolution of implant designs from two-piece to full one-piece PPE frameworks.

The surgical staging of PPE implant auricular reconstruction in managing congenital deformities, including microtia and hemifacial microsomia, has evolved over time. The one-stage approach has become more common, especially when adequate soft tissue coverage is available to cover the implant [12,14,15]. The temporoparietal fascia (TPF) flap remains the workhorse in providing structural support for PPE implants, with reported superior cosmetic outcomes. The surgical timeline is summarized in Figure 2.

Wellisz, Reinisch, Tahiri, and Lewin described their surgical approaches and refinements of this one-stage technique in microtia reconstruction over the last three decades [2,12,15,21]. Nonetheless, for trauma-related and burn cases, surgical staging remained highly variable according to the availability of the surrounding soft tissue envelope and the flap used in auricular reconstruction [2]; see Table 2. Moreover, a significant modification in the temporoparietal facial (TPF) flap was made through periauricular incision, eliminating the need for a scalp incision and reducing the risk of visible scarring and patchy alopecia [8,22]. When necessary, a curvilinear horizontal incision can be extended over the superior portion of the TPF, enhancing the visualization of the distal anterior portion of the superficial temporal artery (STA) [8,22]. In cases requiring further visualization, Z or Y scalp incisions can be attempted [8,22]. On the other hand, the occipital flap serves as an alternative to the TPF flap for patients in whom the TPF is compromised, whether due to initial trauma or its use in an earlier procedure. This flap is elevated through a Z scalp incision over the occipital region after assessing the occipital artery anatomy using Doppler [18].

Notably, few studies collectively examined the integration of different hearing aid techniques in patients with microtia. The implantation of these hearing aids, including traditional external auditory canal meatoplasty, bone bridge (BB), or bone anchored hearing aid (BAHA), can now be performed simultaneously with the PPE auricular reconstruction in a one-stage surgical procedure [23]. Findings indicated that patients undergoing concurrent bone hearing aid implantations experience significant auditory benefits, with mean hearing improvements of 35–43 dB [33]. Conversely, traditional external auditory canal repair remains less effective, yielding suboptimal hearing gains (~4.1 dB) and carrying a higher risk of adverse effects such as canal restenosis or atresia. Importantly, simultaneous hearing aid integration did not lead to the PPE framework’s stability being compromised. Jiang et al. reported that the BB system offered better hearing outcomes over the BAHA, eliminating the risks of chronic infections and skin-related complications [33].

### 3.4. Alternative Flaps When the TPF and Occipital Flaps Are Not Available

In rare cases such as high-impact trauma or severe burn head injuries, identifying a viable flap option can be challenging due to extensive scarring and compromised blood supply. When standard flaps such as TPF and occipital flaps are damaged, the radial forearm free flap serves as a viable option, although it significantly increases surgical staging and complexity. The radial forearm fascia can be transferred as a free flap and used for PPE framework coverage. Radial forearm flap prelamination has also been described, in which a subdermal pouch is created in the volar aspect of the forearm (aligned with the radial artery), serving as vascularized housing for the PPE implant framework. After a four- or six- week period, the radial forearm flap is harvested to the recipient site, ensuring adequate soft tissue coverage of the auricle. While the radial forearm flap remains a potential option in ear reconstruction, it is among the last-resort options due to significant trade-offs, including prolonged PPE surgical staging, possible forearm donor morbidities, and the need for secondary refinements [17,25,29].

#### Two-Stage Approach

**Figure 3:**
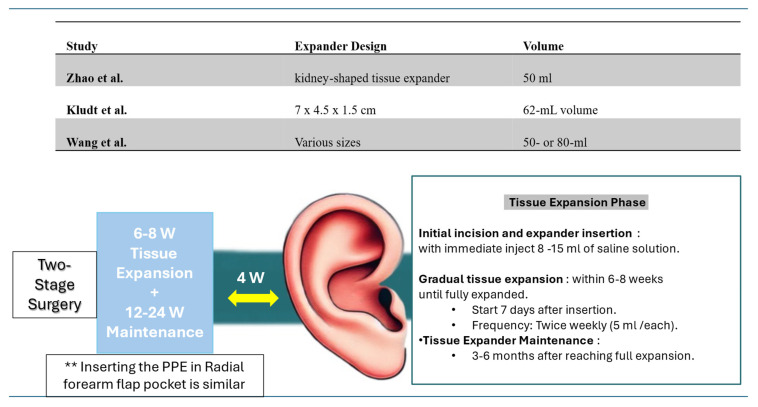
The diagram illustrates key phases of tissue expansion, including expander designs and volumes reported in the included studies [3,20,30].

Although uncommon, some studies have reported the use of tissue expansion prior to PPE implant insertion for various indications, as summarized in Figure 3. This technique, however, extends the surgical timeline, requiring a two-stage or multi-stage approach. This includes 6–8 weeks of tissue expansion followed by 12–24 weeks of maintenance before PPE implant insertion [20].

### 3.5. PPE Framework Evolution

Since the introduction of PPE auricular implants by Berghaus [1,5,9,13,24] and Wellisz [2], the two-piece PPE implant continues to be the predominant framework structure to date. The assembly of the two-piece implant initially involved using warm saline to fuse the external helical rim to the anti-helical base. However, this traditional approach was further refined by Reinisch [12,14,15,16], who enhanced the two-piece PPE implant framework design and extended the base to provide a stronger foundation (Figure 2). Thus, the implant parts are soldered together using a precise handheld cautery intraoperatively. Such an approach has enhanced the connection between parts and reduced the historical rates of implant fractures and extrusions. Recently, Lewin developed a full 1-piece custom auricular implant based on the 3D scan of a usual patient’s auricle, achieving a high anatomical accuracy [8]. As previously highlighted, suitable candidates include children as young as 3–4 years. By this time, the auricle will have reached 85% of its adult size. When reconstruction is performed at this age, the reconstructed auricle is “made slightly larger” to account for the additional growth of the contralateral auricle [7,12].

### 3.6. Reported Complications

The most common reported complication was PPE implant exposure (116/1718, 6.7%) and two partial exposures (less than 1 cm, 0.12%), followed by implant fracture (ranging from 1.6% to 3.2%), infection (12/1718, 0.7%), and wound healing issues (7/1718, 0.4%). The follow up over the studies varied considerably, from 3 months to 19 years (average: 10–35 months). Some minor complications were reported, such as transient hematoma and alopecia, which resolved in 3–4 months. Reinisch et al. documented the largest patient cohort that underwent auricular reconstruction using the PPE implants, comprising 1178 auricles, with reported complications across two different time periods [12]. The complication rates were up to 44% in the early 1990s; however, a recent analysis completed through 2015 indicated a reduction in complications to 8.7% [12]. Thus, complication rates varied due to multiple factors, including surgical technique advances over time and patient-related factors (comorbidities, the congenital deformity grade, and the viability of the surrounding soft tissue envelope). Notably, studies with smaller samples of PPE implants, which showed a greater preference for cartilage grafting, reported higher complication rates. This can be attributed to less experience with alloplastic materials’ surgical techniques. Conversely, larger datasets tended to report complications around 7–13%, suggesting a consistent reduced incidence of major complications with PPE implants (Table 3). Here, complications were classified as major or minor based on the required management approaches. Minor complications are those that could be managed conservatively or with local wound care, while major complications required more surgical intervention.

### 3.7. Management of Complications and Secondary Procedures

Some complications can be managed non-operatively and are expected to resolve spontaneously, such as alopecia and minor wound dehiscence (<1 cm). Hair regrowth was reported to happen in 3–4 months post-operatively. Early hardware exposure should be managed with expedited coverage using local flaps. For prolonged hardware exposure, it is usually beneficial to remove the contaminated framework. Sometimes, a skin graft loss can be seen following the removal of the dressing. If the area of the skin graft loss is less than 1 cm and the underlying flap is viable (i.e., no hardware exposure), epithelialization and uneventful healing can be expected with local wound care. Skin graft loss larger than 1 cm (with viable underlying flap) requires regrafting. Additionally, few studies suggested trimming and contouring of the PPE implants for salvaging the overlying soft tissue coverage. Further reconstructive techniques are recommended when extensive exposures are noticed. Particularly, the co-occurrence of infection and exposure of the PPE implant required a stepwise approach, including extensive wound therapy, antibiotics, and replacement of the PPE implant and even total implant removal. Patients who experienced late implant failure around 1 year often required PPE implant removal and more delayed reimplantation strategies.

### 3.8. Esthetic and Patient-Reported Outcomes

Across the studies, the PPE auricular implants offered superior esthetic results with consistent patient satisfaction. Out of 65 pediatric and adult patients who underwent PPE auricular reconstruction, Braun et al. reported high esthetic satisfaction, with 85% of children and 72.7% of adults satisfied with their reconstructed auricle [34]. Importantly, 100% of children and 75.6% of adults reported improved quality of life postoperatively [34]. The primary complaints of both groups were scarring and the feel of the reconstructed auricle, with patients with acquired auricular defects being twice as likely to be dissatisfied in comparison to those with congenital auricular deformities [34]. Similarly, Wang et al. reported esthetic outcomes, with 70 reconstructed auricles with high patient satisfaction [30]. In their study, PPE implants were associated with a suitable location, stable framework, and smooth skin texture [30]. Lower satisfaction resulted from postoperative complications such as swollen contours and blurry definition, primarily from scar hypertrophy or flap contraction [30]. On the other hand, Constantine et al. conducted a comparative study assessing esthetic outcomes, based on evaluations by blinded observers [26]. In that study, the esthetic outcomes with the PPE auricular reconstruction were compared to those with rib cartilage grafts. PPE implants demonstrated higher scores in shape stability (3.7 vs. 3.2), definition (3.5 vs. 3.1), and framework proportions (4.3 vs. 3.9), but lower scores in color matching (3.9 vs. 4.2) and comparable scores in positioning (4.2 vs. 4.3) [26]. Despite these efforts to quantify esthetic outcomes, the lack of consistent and universally accepted esthetic metrics remains a challenge [2,3,10,11,13,20,22,23,24,29,31,34].

## 4. Discussion

This review of 14 studies comprising 1036 patients revealed promising outcomes for PPE auricle reconstruction since its introduction. Although early surgical approaches in the 1990s were linked to high complication rates (up to 44%) [2,15], complication rates have recently decreased to as low as 7%. In particular, the advancement in temporoparietal fascia (TPF) flap harvesting has been crucial for increased soft tissue coverage viability and decrease implant exposure rates [8,12,15,35]. The success of PPE implants (commonly known as Medpor) in auricle reconstruction is highly dependent on the surgical techniques employed and patient-specific factors [10,14,18,21,22,35]. The PPE implant auricular reconstruction process typically involves a one-stage approach to achieve both esthetic and functional outcomes (Figure 2). Suitable candidates include children as young as 3 years, providing a superior aspect over the conventional autologous cartilage auricle reconstruction. Traditionally, cartilage graft reconstruction requires older children, who are around 10 years old. Several authors have reported favorable outcomes with one-stage procedure when conditions permit immediate PPE framework placement without any need for prior tissue expansion [2,12,15,21]. The harvested flaps and skin grafts will be more than enough to provide an adequate soft tissue envelope. Importantly, PPE framework preparation is reported as an easy but critical step in the success of the auricle reconstruction. This preparation is carried out using warm saline to reshape and smooth the contour of the implants [8,22]. Also, it is recommended to use antibiotics before insertion to minimize infection risks [8,22]. The framework is then secured in place using sutures or suction drains, depending on surgeon preference. Nevertheless, the cornerstone of a successful reconstructive outcome is flap harvesting, with the temporoparietal fascia (TPF) or occipital flaps being common choices. Ensuring flap viability through careful dissection and tunneling helps maintain blood supply and aids in adequate soft tissue coverage. Recently, scalp incisions have been replaced by peri-auricular incisions, enhancing the esthetic component by minimizing the scar burden and reducing the occurrences of patchy alopecia [8,22]. Postoperative care includes silicone molds and suction drains (some surgeons remove drains at the end of the procedure after the application of the silicone mold) to manage healing and prevent complications. Moreover, these procedures can be combined with simultaneous hearing rehabilitation techniques, providing holistic outcomes for patients through external ear meatoplasty, bone bridge, or bone-anchored hearing aids (BAHA) [12,14,33,36]. Thus, the staged approach in auricle reconstruction has been reported to be rare with PPE implants unless there is any necessity for tissue expansion before auricle reconstruction [3,20]. Few studies have reported some minor procedures, including lobular transposition, minor esthetic adjustments, or tragal reconstruction.

### 4.1. Advantages of PPE Auricular Reconstruction

Berghaus et al. highlighted the distinctive architecture of the PPE implants [9], which promote host tissue ingrowth within their pores and thus enhance stability in the surrounding tissue. Although this observation was briefly mentioned in earlier studies, there is a lack of newer studies on this point. Such a knowledge gap has created a silent debate on whether this biological attribute promotes long-term stability or potentially complicates subsequent implant revision or any salvage interventions. Autologous rib cartilage reconstructions, on the other hand, have been extensively researched. Such cartilaginous grafts present challenges, including delayed reconstruction, surgical staging, cartilage absorption, deformation over time, and the additional morbidity of rib harvest; however, they are linked to a lower risk of implant exposure and infection rates [6]. Conversely, PPE auricular reconstruction is a one-stage outpatient procedure that can be performed in children as young as 3 years old. The PPE implant does not carry the risk of long-term warping and resorption. Another advantage is the flexibility in positioning the reconstructed auricle in the desired location, regardless of how low the hairline is, because the hair-bearing tissue can be excised and replaced with a full-thickness skin graft. A low hairline poses a challenge in cases of cartilage-based auricular reconstruction since the majority of the reconstructed auricle is frequently covered with hair-bearing scalp, which detracts from the esthetics of the auricle. Nevertheless, it is important to recognize that despite these PPE advantages, autologous cartilage grafts remain the gold standard in many centers due to their long-standing clinical track record, surgeon familiarity, and robust literature support, particularly in high-risk cases where long-term integration and minimal foreign body response are prioritized.

### 4.2. Global Adoption Trends of PPE Auricular Reconstruction

**Figure 4:**
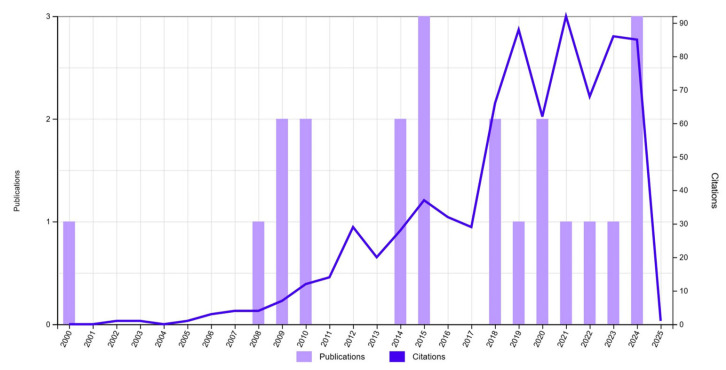
Web of Science citation record of the PPE implant literature in auricular reconstruction, illustrating the number of publications and corresponding citation counts over time.

Although autologous rib cartilage grafting remained the gold standard method over the last century, PPE implants have shown a significant peak in publications and citations recently. This surge likely corresponds to the increased dissemination of results by leading surgeons who advocate for PPE implants and have contributed to refinements in surgical techniques and advancements in the PPE framework designs (Figure 4). This trend can also be attributed to the improvements in imaging modalities like 3D scanning and assisting technologies and the microsurgical flap harvesting techniques. Thus, many surgeons have evaluated the risk–benefit ratio and started to integrate the innovative PPE technique in their auricular reconstruction practice, thereby avoiding the additional site morbidity and advocating for an early primary repair option with a single-stage procedure in congenital auricular deformities.

Despite advances in PPE implant adoption in high-resource settings, its adoption in low- and middle-income (LMIC) countries remains limited [7]. The geographic distribution in Figure 1 underscores a significant gap in the literature, and particularly the lack of comprehensive cost-effectiveness analyses. Within under-resourced health systems, PPE implementation is hindered by limited access to enabling technologies and continuity of care, including 3D scanning, digital workflow integration, and specialized surgical training. Inconsistent or unavailable longitudinal follow-up also hinders global PPE adoption, particularly in light of previously reported complication rates over the past two decades. As elaborated earlier, while PPE auricular reconstruction provides advantages such as fewer surgical stages and the possibility of single-stage outpatient procedures, its substantial upfront cost remains a key barrier—though it may be offset by downstream efficiencies over time. Addressing such technological, educational, and infrastructural limitations is essential to achieving equitable global access to advanced auricular reconstruction techniques.

Additionally, a recent study by Reinisch et al. examined 144 cases of secondary auricle reconstruction to address failures from initial procedures, including 91 cases with rib cartilage, 47 with PPE implants, 4 with prostheses, and 2 with cadaver cartilage [17]. All secondary procedures utilized PPE implants due to their low morbidity and suitability for single-stage, outpatient surgeries [17]. Complications were observed in 10% of cases, mainly involving minor framework exposure or partial flap failure, and were successfully managed with local revisions [17].

Compared to rib cartilage grafts, PPE implants have demonstrated superior esthetic outcomes in shape stability, definition, and framework proportions, and achieved similar positioning, but were slightly inferior in color matching [26]. While prior studies have reported high esthetic satisfaction (70–85%) following PPE reconstruction [34], the lack of standardized outcome measures limits comparability across cohorts. Recent efforts to develop validated patient-reported outcome measures, such as the EAR-Q, aim to address this gap by systematically capturing domains relevant to individuals with ear conditions, including appearance, adverse effects, and psychosocial impact [37]. Furthermore, newly established normative data in healthy adult populations enhance its interpretability in clinical and research settings [38]. Lastly, the interdisciplinary approach, combining the expertise of plastic surgeons, otolaryngologists, and audiologists, is essential for achieving comprehensive care that addresses both the esthetic and functional needs of patients. Overall, PPE implants have demonstrated efficacy in both primary auricular reconstruction and secondary salvage. Therefore, ongoing advancements in implant materials, along with refining techniques for flap harvesting and soft tissue coverage, have contributed to improved postoperative outcomes [3,20,25,39,40,41,42,43].

## 5. Limitations

This review was limited by the paucity of the studies reporting the use of PPE implants in auricle reconstruction. The PPE auricular implants remain a small fraction compared to the widely used autologous rib cartilage grafting. While most of the included studies reported short-term and long-term outcomes, the overall quality of evidence remains inconsistent, with variations in study design, follow-up duration, and outcome measures. Some PPE auricular reconstruction cases have been reported within a substantial cohort of patients who underwent alternative approaches for auricle reconstruction. Additionally, some studies followed unclear reporting metrics regarding the number of procedures, auricles, and the patients; some reported numbers, while others provided percentages. When the same research group publishes follow-up studies, it becomes unclear whether the recent data represent new cases’ outcomes or re-evaluations of previously reported patients. This consequently complicates the capacity to draw any conclusions on the longevity of the PPE auricular reconstruction. However, this approach may have introduced an element of selection bias. These limitations highlight the need for well-designed, prospective clinical studies with standardized reporting and long-term follow up metrics to ensure reliable and reproducible outcomes in PPE auricular reconstruction. In these, a consensus should be made on what constitutes major and minor complications for the better analysis of the surgical success rates and PPE implant longevity. We encourage future studies on auricular reconstruction to incorporate the EAR-Q to enhance the evaluation of patient-reported outcomes. Additionally, clinical research should incorporate comprehensive economic evaluations to determine the sustainability and scalability of PPE reconstruction worldwide.

## 6. Conclusions

Although porous polyethylene auricle implants have been in use for nearly half a century, their global adoption remained limited due to the paucity of publications and fear of complications and longevity. These 3D implants offer a promising surgical reconstruction option, allowing for early intervention, favorable esthetic results, and the elimination of donor-site morbidity linked to rib cartilage harvest. Recently, the reported complication rates, including implant exposures, infections, and fractures, have been considerably lowered by improvements in surgical techniques, such as improved flap harvesting and skin coverage techniques. Implant exposures in well-executed instances can be as low as 7%. More in-depth clinical studies to provide long-term evidence are encouraged.

## Figures and Tables

**Table 1:** Summary of patient demographics and surgical indications from included studies, including number of auricles and patients, age ranges, gender distribution, reconstruction indications, and types of PPE implants used.

	Results	Range	Average
Auricles and Patients	1718 auricles for 1036 patients		
Reconstruction indications	Congenital deformities: microtia/anotia, hemifacial microsomia, Cosmon cleft auricles deformity, burn, and trauma		
Age (in years) ^ Children: Adults:	__	3 years old to 14 years old 20 years old to 59 years old	8.2 years old 31.7 years old
Gender ^ Female Male	83 52	Some studies reported gender category in percentage: 64% female and 36% male.	_
PPE implant used	Medpor (Porex Surgical, Newnan, GA, USA) Medpor (Stryker, Kalamazoo, MI, USA)		

^ The age and gender reports varied across studies.

**Table 2:** List of the included articles, outlining study design, sample size, study duration, surgical indications, number of stages, and flap types used for auricular reconstruction.

Authors	Year	Country	Study Design	Sample Size (Patients)	Duration of Study (From-To)	Surgical Indication	Surgical Stages	Flap Type
Wellisz et al. [2]	1993	USA	Retrospective	26 auricles for 18 patients	May 1988 through May 1992	Burn	2	TPF
Romo et al. [23]	2009	USA	Prospective	28 auricles for 25 patients	2000 through 2006	Microtia-atresia (grade III microtia with complete bony EAC atresia); 14 patients right atresia, 8 left atresia, and 3 bilateral atresia	2	TPF
Zhao et al. [3]	2009	China	Retrospective	355	2002 through January 2006	Majority is microtia, post burn or trauma	2	TPF
Berghaus et al. [24]	2012	Germany	Case report	1 auricle	2012	Cosman cleft auricle deformity	1	Postauricular fascia flap
Simsek et al. [25]	2012	Turkey	Case report	1 auricle	2011	Traumatic amputation of the left auricle	2	Radial forearm flap
Kludt et al. [20]	2014	USA	Case series	15 patients	2014	All with either grade 3 or 4 microtia.	3	TPF
Constantine et al. [26]	2014	USA	Retrospective	17 auricles for 17 patients	2001 through 2012	Microtia	1	TPF
Reinisch et al. [12]	2015	USA	Retrospective	1178 auricles (earlier: 25 procedures)	March 1991 through September 2015 *1993–1995*	(62.9%) had no atresia repair, 211 (22.0%) had a prior atresia repair, and 144 (15.0%) had an atresia repair at the time of the auricular reconstruction. Bilateral microtia in 11.2%, 603 Initially, 2 (ear framework then concha and tragal reconstruction).	_	0
				1178 auricles (recent: 487 procedures)	March 1991 through September 2015 *2008–2013*		Mostly 1 stage	Mainly TPF
Fernandes et al. [27]	2016	USA	Retrospective	17 auricles for 16 patients	2004 through 2012	Burn	2	TPF
Chen et al. [28]	2017	Taiwan and Singapore	Prospective	6 auricles for 6 patients	January 2015 through January 2016	Unilateral microtia with hemifacial microsomia	0	TPF
Horta et al. [29]	2018	Portugal	Case report	1 auricle	2018	Traumatic Amputation	2	Radial forearm Flap
Wang et al. [30]	2021	China	Retrospective	70 auricles for 68 patients	1998 through 2018	0	0	Expanded skin flap, and TPF or postauricular fascia
Bini et al. [31]	2024	Greece	Case Report	1 auricle	2024	Right hemifacial microsomia and anotia	1	TPF
Gomez et al. [32]	2024	USA	Case reports	2 auricles for 2 patients	2024	grade III microtia with atresia and left grade III	1	TPF

**Table 3:** List of the included articles detailing reported postoperative complications associated with auricular reconstruction techniques, along with the type and extent of interventions required for management.

Authors	Sample Size (Patients)	Reported Complications	Follow-Up Period	No. of Major Complications	No. of Minor Complications	Intervention Required for Complication
Wellisz et al. (1993) [2]	26 auricles for 18 patients	2 exposures (after 4 weeks and 6 weeks); non-patent superficial temporal vessels with eschar 1 × 2 cm, and the second due to lack of complete coverage with the flap, at 6 weeks	2 years (mean 10 months)	2	0	At 4 weeks a split thickness skin graft was applied, the second exposure, trimming of the implants
Romo et al. (2009) [23]	28 auricles for 25 patients	7 cases of minor complications, 6 wound dehiscence (<1 cm), from trauma to the region in the postoperative period. There was 1 postauricular hematoma, which was aspirated in the office.	6 to 60 months (mean 35 months).	0	7	2 cases further touch-up work (scar revision)
Zhao et al. (2009) [3]	355	48 cases of exposures and 1 infection	3 months to 5 years	48	1	Not specified
Berghaus et al. (2012) [24]	1 auricle	0	6 months	0	0	_
Simsek et al. (2012) [25]	1 auricle	0	1 year	0	0	_
Kludt et al. (2014) [20]	15 patients	1 exposure	from 6 months to 5 years	1	0	Exposed implant was resected and covered with a random flap
Constantine et al. (2014) [26]	17 auricles for 17 patients	2 (1 infection and 1 extrusion)	from 2 to 6 years	2	0	Underwent subsequent reconstruction with cartilage grafts
Reinisch et al. (2015) [12]	1178 auricles	Of 25 procedures, there were 7 implant fractures, 11 exposures, and 1 infection	3 years (min)	18	1	Not specified
		Of 487 procedures, there were 7 to 42 implant fractures, 21 exposures, and 5 infections	1.5 year (min)	~46	5	Not specified
Fernandes et al. (2016) [27]	17 auricles for 16 patients	2 exposures	up to 5 years.	2	0	Replacement of the PPE implant with local advancement flap, and the other exposure removal with primary closure
Chen et al. (2017) [28]	6 auricles for 6 patients	2 transitory alopecia and 1 partial exposure	10.3 months	0	1	The hair grew up 3 to 4 months
Horta et al. (2018) [29]	1 auricle	0	3 to 6 months	0	0	0
Wang et al. (2021) [30]	70 auricles for 68 patients	16 auricles in 15 patients presented with complications (22.06%), including 9 framework exposures (13.24%), 3 infections (4.41%), 2 scar hypertrophy (4.41 %), and 2 hematomas (2.94%)	6 months to 19 years.	9	8	Frameworks were taken out due to intractable exposure
Bini et al. (2024) [31]	1 auricle	Partial exposure due to inflammation and infection	10 months	1	0	A temporalis muscular flap along with the deep temporal fascia were used as a salvage operation and a full thickness skin graft. Auricular helix reconstruction was completed with a rotation scalp flap after tissue expansion
Gomez et al. (2024) [32]	2 auricles for 2 patients	One-by-one centimeter area along the distal posterior helix was noted to have implant exposure. Ten weeks post-operatively the patients presented with copious purulent discharge from a small (one by two millimeter) area of implant exposure along the inferior aspect of the retro-auricular sulcus.	6 months to 12 months	2	0	Antibiotics and primary wound closure, plus one week of negative pressure wound therapy (NPWT), and the other patient underwent a PPE implant replacement

## Data Availability

Not applicable.

## Supplementary Materials

The following supporting information can be downloaded at https://www.mdpi.com/article/10.3390/jcm14124116/s1, Figure S1: PRISMA flow diagram summarizing the study selection process for articles on ear reconstruction using porous polyethylene (PPE) implants.

## Abbreviations

The following abbreviations are used in this manuscript:

PPE	Porous polyethylene
TPF	Temporoparietal fascia
