# Supplementary material for: Advancing Auricular Reconstruction: The Evolution and Outcomes of Auricular Reconstruction Using a Porous Polyethylene (PPE) Framework
Source: J Clin Med. 2025 Jun 10;14(12):4116. doi: 10.3390/jcm14124116 (PMC12194376; doi:10.3390/jcm14124116)
Supplement: Supplementary file 1 [file jcm-14-04116-s001.zip › jcm-3611736-supplementary.pdf]

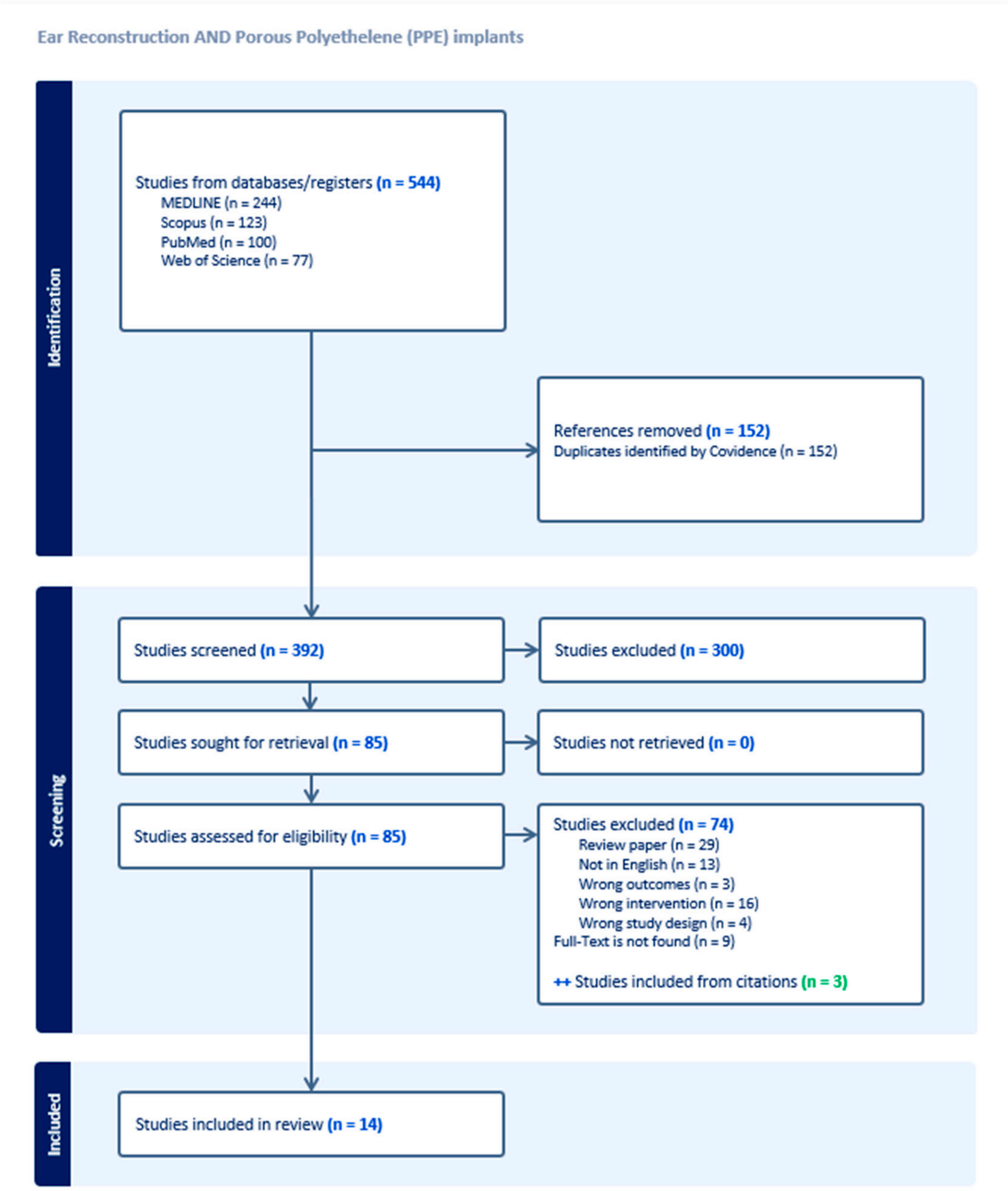

Figure S1. PRISMA flow diagram summarizing the study selection process for articles on ear reconstruction using Porous Polyethylene (PPE) implants.
